# Fatal Errors

**Published:** 1890-08

**Authors:** J. H. Jackson

**Affiliations:** Flint, Mich.


### FATAL ERRORS.

J. H. Jackson, M. D., Flint, Mich.

"Don't hold a penny so close to your eye that you can't see a dollar a little further off," was the advice I once heard a friend give one of his agents.

The besetting sin of many who *pretend* to follow Hahnemann is that they hold a pathological lesion so close to their eyes that they fail to see the individuality of their patients.

If a patient should consult a physician of this class for treatment for an epithelial cancer of right ala nasi, the probability is that he would prescribe Kreosotum, *because* it has epithelial cancer of that locality.

He would not think that in the first place whoever gave Kreosotum for such a lesion probably prescribed it on account of other symptoms that were covered by the remedy, and as the *symptom*, *not* disease, epithelial cancer disappeared under the use of Kreosotum, the *symptom* epithelial cancer of right ala nasi has become recorded under this remedy. Therefore to prescribe it for this pathological symptom alone would *not* be a proper way to proceed in the treatment of the sick.

If a cure followed it would be an accidental one, as a pathological lesion is simply an outward manifestation of an *internal* contamination, and of itself many times unsufficient basis for a homœopathic prescription.

I was once called to treat a boy for phymosis. On page 68 of *Berjeau on Syphilis*, under Merc-cor., can be found these words: "Indicated when there is violent swelling of prepuce like a bladder, with burning, biting, redness, and painful sensibility, with cracks and fissures on inner surface." This was an exact description of the local symptoms which, in this case, might have been abridged to have read—phymosis, for all the good they would have been as a guide for the selection of the most truly homœopathic remedy. For in addition to the local symptoms the child's urine would become like milk after standing awhile; the cheeks were very red, with well-defined bluish-whiteness about mouth and nose. He could not bear you to look at him, and to touch him was the signal for an outbreak of rage and scolding. His mother said that in sleep he was constantly grating his teeth, and boring at and rubbing his nose in sleep or awake.

Now, what was the correct way to proceed in order to heal this patient in a truly Hahnemannian manner? Give Merc-cor. because it covered the pathological lesion, or Cina, which corresponds with the patient's individuality?

It would have been a fatal error to give Merc-cor., for it was *not* homœopathic to the case, but Cina *was*, even though it had never been known to cause or cure phymosis. Therefore Cina$^{1m}$ was administered, and was followed by amiability of temper and speedy recovery of health.

Suppose I were to write a materia medica, and under Cina should record, "Phymosis, violent swelling of prepuce like a bladder, with burning, biting, redness, and painful sensibility, with cracks and fissures on inner surface." It would indeed be a fatal error for a physician who might have a case of phymosis presenting an exactly similar pathological state to hastily give Cina, without first scanning the case for mental symptoms and all other objective and subjective signs that, in their entirety, voice the true picture of a case of sickness, and infallibly demand for its removal *that* remedy capable of producing exactly similar symptoms to the most *striking* and *peculiar*, therefore most *characteristic* symptoms of the patient.

The mental symptoms, "cannot bear to be looked at, nor touched," were very striking and peculiar, and, on that account, ought always to overshadow common or ordinary symptoms.

It matters not whether a tumor of the breast following a blow had ever been cured by Chamomilla or Cina, if the mental and other symptoms of the patient were covered by either of them, it would be in accord with strict Homœopathy to give either of them, and not the routine remedy, Conium.

It is a fatal error to give "usual" remedies, *because* of their being usual remedies, or ones most frequently indicated. A large percentage of failures is due to routine prescriptions, particularly by the mongrels, who never have allowed the beautiful truths of our divine art to permeate their very souls. I say allowed—the majority of them are probably incapable of becoming in harmony with truth, as they are hopelessly infatuated by the tinsel and trash of the "physiological livery."

A woman presenting in her face what the books describe as the cancer-cachexia, consulted me about a tumor involving the right labia majora and for a distance of six inches down inner surface of thigh, and deeper tissues for about three inches, the growth being about three inches wide, and probably weighing a pound or pound and a half.

The tumor being of stony hardness, had taken a year to attain its present proportions.

The labia majora was everted and hard as a stone, the patient complained of sharp, plunging pains in tumor, worse after handling it, and at night.

The woman's mother was said to have died of cancer of stomach at age of forty. The patient was thirty-nine. She told me that a homœopathic surgeon had pronounced her tumor a cancer, and said that the only remedy was the knife.

Several allopathic surgeons had given the same opinion.

I told the poor creature that remedies might cure her. The first examination elicited these symptoms: burning of feet in bed, must put them from under the covers; swelling of the feet so that much of the time cannot wear shoes; hot flashes upward, followed by great faintness and sweat, top of head so burning hot is compelled to keep it drenched with cold water; marked faintness at eleven A. M.; at times diarrhœa in early morning. Sulphur$^{cm}$ one dose, and Sac-lac. three times a day for two weeks.

On second examination, I was informed that all the symptoms, except lancinating pains in tumor, had improved for about eight days, when they returned and were now about same as at first. Another dose of Sulphur$^{cm}$ was given and Sac-lac. for two weeks.

At third examination she told me that all symptoms had ceased, except pains in tumor, which were *very* sharp, and that there were *sharp, plunging pains* back of ears, in arms, in abdomen, and in various parts of body, and that her feet do not burn, but are more swollen than ever.

Examination of tumor revealed no perceptible change.

Now, sharp, lancinating pains are characteristic of a scirrhus, and are, on that account, *not* a characteristic or guiding symptom. But sharp, lancinating pains in various parts of the body of a person afflicted by cancer, *are* striking and peculiar, and, therefore, characteristic, and as Apis-mel. has prominently these pains, and as it covers the œdema of feet and thirstlessness and scanty urine, which she now complains of, $Apis^{cm}$ was given, with a request to report in one week.

When she came to see me she said that after taking the first dose of medicine, the sharp pains were more intense than ever and more frequent, and that where the pains came, would be sore for hours after.

After the aggravation of pains passed off, the swelling of feet, scanty urine, and thirstlessness were improved for a few days, but now are about the same.

A second dose of Apis was given and in a week's time she entered my office with the exclamation, "O doctor! I think there is a change in the swelling." Examination of it revealed the fact that the tumor seemed to have broken up into angular pieces that were very hard to the touch and could be felt to move slightly on manipulation. *No medicine.*

In two weeks I again examined tumor and found the angular pieces could be plainly felt and that they were freely movable about, the labia majora was also less everted. The patient now complains of passing large quantities of colorless urine. *Her stomach seems full from a few mouthfuls* of food, and has great distress from gas in stomach and abdomen and rumbling in abdomen and in left hypochondriac region. Prescribed $Lycopodium^{cm}$, one dose. In one week reports that the remedy seemed to act like magic, as she was relieved of symptoms at once, but that for the last day or so has had some return. Lycopodium$^{cm}$, one powder, and in a week another dose, and these *seven* doses of medicine in the CM potency were all the medicine this woman received, and in two weeks from giving the last dose *not a vestige of the tumor remained*, and the patient presented a perfect picture of health.

Who can contemplate such a marvelous proof of the truth of the law of the similars, and not love the man who was so open to divine influx as to receive into the chamber of his radiant mind so full and perfect a revelation of the only art of healing worthy the name?

And who among us that has plodded through many a weary hour of faithful search of the materia medica, that we might do honor to our loved art, and do good to those who were sick, and who *know* that this alone is the royal road to "restore health to the sick, which is called healing," can have other than scorn and contempt for the barnacles that "trade upon a name"? Who deceive and lie and slander and slay that they may make money and escape the labor that alone enables one to do the best possible for the sick and suffering.

When we, who know the better way, witness the fearful mortality attending the allopathist's art of bungling, and the little better that attends the ministrations of the mongrels, and in addition, the worse than death—the various torturing mental states that make the lives of hundreds of thousands a very hell: the frightful wastings, the excessively fat, and the walking skeletons, the distorted, lame, halt, misshapen, and blind: the everted fiery red eyelids, burned "scientifically" by caustics till the human countenance divine is loathsome to behold: the corneal opacities that shade forever the windows of the soul: the slow death by inches by consumption, and by cancers that eat and eat and burn like fire, and produce a stench that makes the abode of the sufferer a charnel-house of horror: the thousands that throng the mad-houses, *sent* there by as vile and reprehensible a mal-practice as ever disgraced the veriest ignoramus, a mal-practice perpetrated by graduates of a "regular" school, established to teach and train men in that most fearful art of violating nature's laws, done in the name of "science"—

*regular* science: the thousands that are sent into the world but half made up, idiots, imbeciles, blind, deaf, and dumb; their rounding out and harmonious development blighted in their mother's womb by that blasphemy against truth and common sense and humanity, regular and mongrel methods of treatment that violate the law of metastasis in the treatment of the complaints of women during the stage of gestation, and, consequently, abort the mental or physical development of the fœtus: when we witness these fearful consequences, it is well to denounce in no uncertain voice the vile mongrel who deceives and misleads and does more harm to a propagation of pure Homœopathy than many true followers of Hahnemann can overcome in a lifetime.

We all ought to have charity for honest error, *but it is a fatal error not to denounce criminal negligence,* and it is nothing short of criminal negligence for professed homœopathists to resort to "cobbling" allopathic methods of *suppressing* manifestations of diseased action, instead of studying the materia medica and becoming thereby competent healers of the sick.

